# Differences in left ventricular strain measurements between cine DENSE cardiac magnetic resonance and SSFP feature tracking

**DOI:** 10.1186/1532-429X-18-S1-O76

**Published:** 2016-01-27

**Authors:** Christopher M Haggerty, Jared A Feindt, Dimitri Mojsejenko, Gregory J Wehner, Jonathan D Suever, Mark A Fogel, Brandon K Fornwalt

**Affiliations:** 1 grid.415341.60000000404334040Geisinger Clinic, Danville, PA USA; 2 grid.253363.20000000122979828Bucknell University, Lewisburg, PA USA; 3 grid.266539.d0000000419368438University of Kentucky, Lexington, KY USA; 4 grid.239552.a0000000106808770Children's Hospital of Philadelphia, Philadelphia, PA USA

## Background

Left ventricular (LV) mechanics (e.g., strain) provide a clinically relevant description of LV function. Automated endocardial feature tracking (FT) for steady state free precession (SSFP) images is increasingly used to quantify strain, yet accuracy limitations have been noted. Furthermore, similarities between FT and global strain calculated from endocardial contour lengths have been noted, but the contribution of epicardial contour strain are unknown. Displacement encoding with stimulated echoes (DENSE) CMR measures myocardial motion and thus provides a gold standard to evaluate these differences. We hypothesized that FT or endo-/epicardial contour strains would not agree with DENSE strains.

## Methods

We reviewed our CMR database to identify instances in which SSFP images and DENSE were acquired at the same location. Additional data meeting this criterion were prospectively acquired at the Children's Hospital of Philadelphia. Collectively, 93 image pairs (66 short-axis, 27 long-axis) were included from 25 volunteers (23 ± 17 yrs) and 18 patients with heart disease (17 ± 24 yrs), all of whom consented for research. Commercial FT software (TomTec Imaging Systems) was used to semi-automatically track endocardial motion in the SSFP images. The endo- and epicardial boundaries were also manually traced at the end-diastolic and end-systolic frames to compute contour (Green) strain using custom MATLAB software. DENSE images were post-processed in MATLAB. Average peak circumferential and longitudinal strains were compared across techniques using repeated measures ANOVA and 95% limits of agreement (LoA).

## Results

For circumferential strain, contour-derived values significantly (p < 0.001) overestimated DENSE measures (Table). FT and endocardial contour strain had mean biases of 9% and 8%, respectively. The data were also highly variable, with wide 95% LoA (Table, Figure). Only the averaged endo- and epicardial contour strains demonstrated marginal agreement with DENSE. Conversely, there was no statistical difference between FT and endocardial contour strain (p = 0.26, LoA= ± 7%). For longitudinal strain, agreement between FT and DENSE was slightly improved--there was only a non-significant trend toward larger FT strain (p = 0.08)--but substantial variability was still present (LoA= ± 9%; Figure). Both contour strain estimates had large biases compared to DENSE, but small variability (LoA= ± 5%).

## Conclusions

Average circumferential strain is poorly approximated using endocardial contours as they over-estimate the measured result with substantial variability. Incorporating epicardial contours into feature tracking assessments may improve accuracy, although defining average strain from contour length changes--without tracking--yields similar results. Feature tracking for longitudinal strain is more accurate than circumferential measurements, but is still highly variable compared to DENSE. These differences could confound attempts to establish universal prognostic thresholds for strain patterns in disease.

**Table 1:** Table 1

	Circumferential strain [%]	Longitudinal strain [%]	95% LoA_SA_ Range [%] (compared to DENSE)	95% LoA_LA_ Range [%] (compared to DENSE)
DENSE	17 ± 4	14 ± 3	N/A	N/A
FT	26 ± 6	16 ± 4	± 12	± 9
Endocardial contour	26 ± 5	21 ± 3	± 10	± 5
Endo- and Epicardial contours	19 ± 4	19 ± 3	± 9	± 5

**Figure 1:**
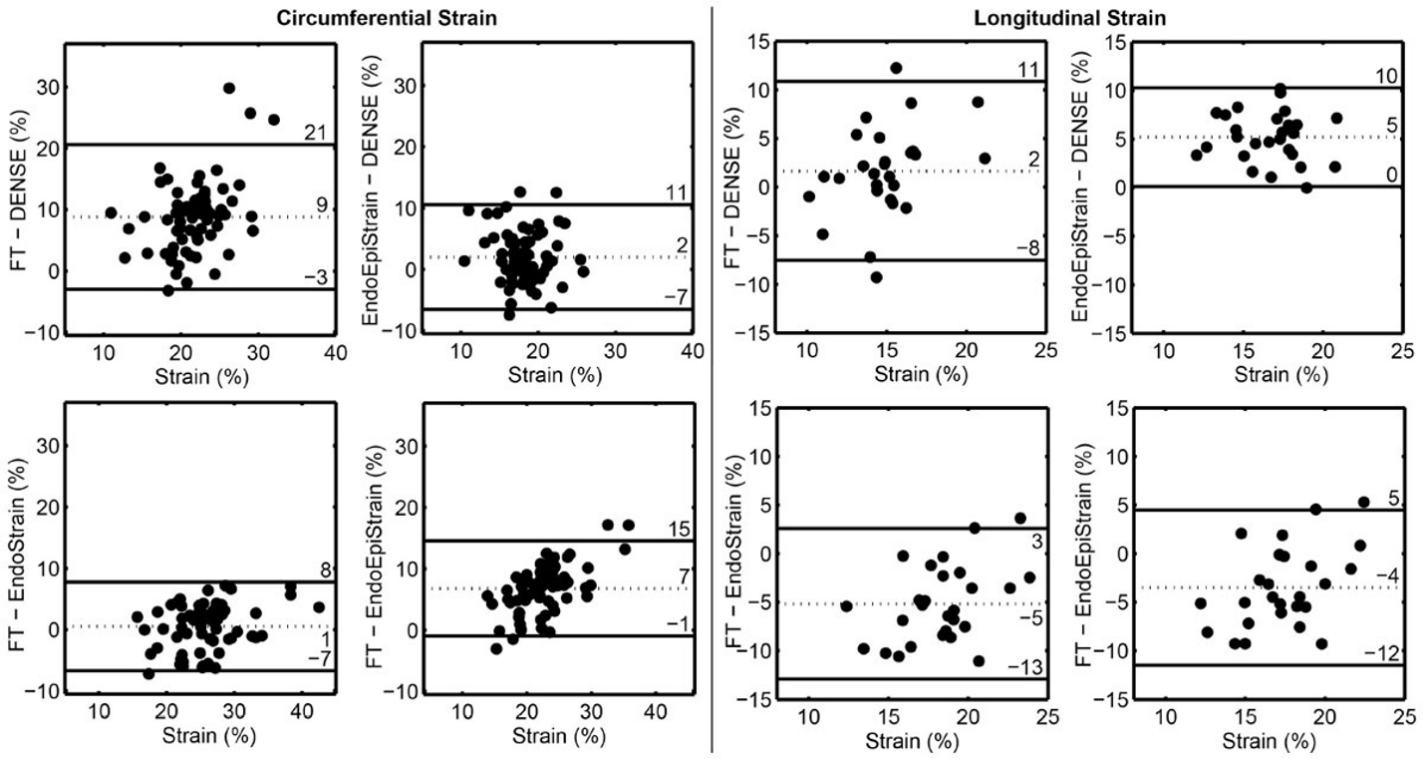
**Limits of Agreement comparisons between techniques for average circumferential (left pane) and longitudinal strains (right pane)**.

